# Mechanistic evaluation of antiarthritic and anti-inflammatory effect of campesterol ester derivatives in complete Freund’s adjuvant-induced arthritic rats

**DOI:** 10.3389/fphar.2023.1346054

**Published:** 2024-01-23

**Authors:** Sarwat Nazir, Ishtiaq Ahmad, Aisha Mobashar, Ali Sharif, Arham Shabbir, Waqas Ashraf Chaudhary

**Affiliations:** ^1^ Department of Pharmacology, Faculty of Pharmacy, The University of Lahore, Lahore, Pakistan; ^2^ Department of Global Health Research, Graduate School of Medicine, Juntendo University, Tokyo, Japan; ^3^ Department of Pharmacology, Institute of Pharmacy, Faculty of Pharmaceutical and Allied Health Sciences, Lahore College for Women University, Jail road, Lahore, Pakistan; ^4^ Department of Pain Management, School of Biological Sciences, University of Leicester, Leicester, England

**Keywords:** arthritis, anti-inflammatory effects, campesterol ester derivatives, complete Freund’s adjuvant, Sprague Dawley rats

## Abstract

**Background:** Current therapies for RA have limitations and side effects, leading to a growing need for safer treatment options. Natural compounds from plants are gaining attention for their therapeutic benefits and fewer side effects. One such compound is the campesterol derivative, a steroid derivative occurring in plants. Studies have shown that this derivative has anti-inflammatory properties and can impact the expression of pro-inflammatory factors. The primary objective of this study was to explore and assess the potential therapeutic effects of Campesterol Ester Derivatives (CED) utilizing a rat model of arthritis induced by Complete Freund’s Adjuvant (CFA).

**Method:** The rats were divided into specific experimental groups and treated with either CED or piroxicam (as a positive control) for a duration of 28 days. We determined the effects of CED on various parameters including paw edema, thermal hyperalgesia, and mechanical allodynia at different time points. Furthermore, serum levels of inflammatory cytokines, oxidative stress markers and histological analyses were performed. Additionally, mRNA expression levels of inflammatory markers, both pro-inflammatory (such as TNF-α, NF-κB, IL-6, COX-1, COX-2, and IL-4) and anti-inflammatory were analyzed.

**Results:** In the arthritic rat model, CED exhibited significant anti-inflammatory effects and resulted in a notable reduction in paw edema levels compared to the control group. Histopathological examination of the treated rats’ paws confirmed a decrease in inflammation and tissue damage, including reduced pannus formation and bone erosion. Importantly, there were no observable signs of damage to the liver and kidneys following CED treatment, indicating its safety profile and potential for organ protection. At the molecular level, CED treatment downregulated mRNA expression levels of pro-inflammatory markers, indicating its ability to suppress inflammation. Conversely, certain anti-inflammatory markers were upregulated following CED treatment, suggesting a positive influence on the immune response. The positive effects of CED were not limited to joint inflammation; it also showed systemic benefits by positively influencing hematological and biochemical parameters.

**Conclusion:** CED demonstrated promising therapeutic potential as an anti-inflammatory intervention for arthritis in the experimental rat model. Its ability to reduce inflammation, protect tissues, and improve organ function indicates its multifaceted benefits.

## 1 Introduction

Nature’s creation is intricately organized, but disruptions can occur, such as the immune system’s aggressive reaction against self-antigen cells in autoimmune disorders. Among these disorders, Rheumatoid Arthritis (RA) stands as a prominent example. RA is a chronic and progressive autoimmune disease affecting many individuals worldwide (Radu and Bungau, 2021; Guo et al., 2018). Additionally, it imposes a considerable economic burden on healthcare systems and society. Despite notable strides in medical science and therapeutic options, the challenges posed by RA persist. Both patients and healthcare professionals continue to grapple with its complex and multifaceted nature. Further research and advancements are essential to enhance the management and care for individuals affected by this autoimmune condition (Finckh et al., 2022).

The pathogenesis of RA remains complex and multifactorial, while the exact etiology of the disease is not fully understood, it is widely believed to arise from the interplay of genetic predisposition, environmental triggers, and dysregulated immune responses (Aletaha and Smolen, 2018). The immune system’s involvement in RA is characterized by a cascade of events involving various cell types, cytokines, chemokines, and autoantibodies. T-cells and B-cells play pivotal roles in the autoimmune responses that target the synovium, leading to chronic inflammation and subsequent joint destruction. Pro-inflammatory cytokines such as interleukin-6 (IL-6) and tumor necrosis factor-alpha (TNF-α) are key players in driving the inflammatory process and perpetuating tissue damage (Brennan and McInnes, 2008; Choy, 2012).

Current treatment approaches for RA include nonsteroidal anti-inflammatory drugs (NSAIDs), disease-modifying anti-rheumatic drugs (DMARDs), and biologic agents that target specific inflammatory pathways (Abbasi et al., 2019). While these treatments have significantly relieved many patients, they are not without limitations. While effective in reducing pain and inflammation, NSAIDs can cause gastrointestinal bleeding and other adverse effects (Bindu et al., 2020). Although effective in slowing disease progression, DMARDs may lead to immune suppression, making patients susceptible to infections. Biologics, while highly specific and effective, can be costly and not affordable for all patients (Brennan and McInnes, 2008; Joensuu et al., 2015).

Given current therapies’ limitations and potential side effects, there is a growing need to explore novel and safer treatment options for RA. Natural compounds derived from plants have gained attention recently due to their potential therapeutic benefits with fewer adverse effects (Li et al., 2017). One such compound is the campesterol derivative, a steroid derivative naturally occurring in plants. Previous research has shown that this derivative exhibits anti-inflammatory properties and can modulate the expression of pro-inflammatory factors (Lin et al., 2018). These properties make it an intriguing candidate for investigating its potential as an alternative or adjunct therapeutic agent for RA.

In light of the above considerations, this research study aims to evaluate the mechanistic effects of the campesterol derivative in an experimental rat model of RA. The study seeks to assess the compound’s ability to ameliorate symptoms of arthritis, reduce inflammation, and protect joint structures. By understanding how the campesterol derivative influences the immune and inflammatory pathways associated with RA, we hope to provide valuable insights into its potential as a novel therapeutic approach for this challenging autoimmune disorder.

## 2 Materials and methods

### 2.1 Animal model and experimental design

Sprague Dawley rats (SD-FNTac: SD and SD-MNTac: SD genotype) were used as the experimental animals in this study owing to their capability to exhibit excellent reproductive performance, making them suitable for generating timed pregnant females. The rats were maintained as an outbred closed colony throughout the study. Adult female Sprague Dawley rats weighing between 250 and 350 g were used in the experiments. The animals were fed with NIH #31M rodent diet and had *ad libitum* access to food and water. The rats were housed in standard laboratory conditions with a 22°C–25°C temperature and a 12:12 h light-dark cycle.

### 2.2 Induction of arthritis and treatment protocol

The Arthritis was induced in the rats on day 0 by administering a 0.1 mL hypodermic injection of Complete Freund’s Adjuvant (CFA) into the right hind footpad. The samples were then divided into specific experimental groups, and all drugs or the vehicle control (CED dissolved in saline with s10% Tween 80 were accurately administered through oral gavage. Group I served as the control, where rats received 0.1 mL of normal saline instead of CFA in the right hind paw. In Group II, rats were given 0.1 mL of CFA in the right hind paw to induce arthritis. Group III, the positive control, received piroxicam (10 mg/kg, PO) daily for 21 days following CFA injection. Rats in Group IV were treated with CED (50 mg/kg, PO) daily for 21 days after the CFA injection, while Group V received a higher dose of CED (100 mg/kg, PO) daily for 21 days following the CFA injection. Measurements of Paw volume was measured by a plethysmometer at 0, 8, 15, 22 and 28 days after induction.

### 2.3 Assessment of arthritis

**FIGURE 1:**
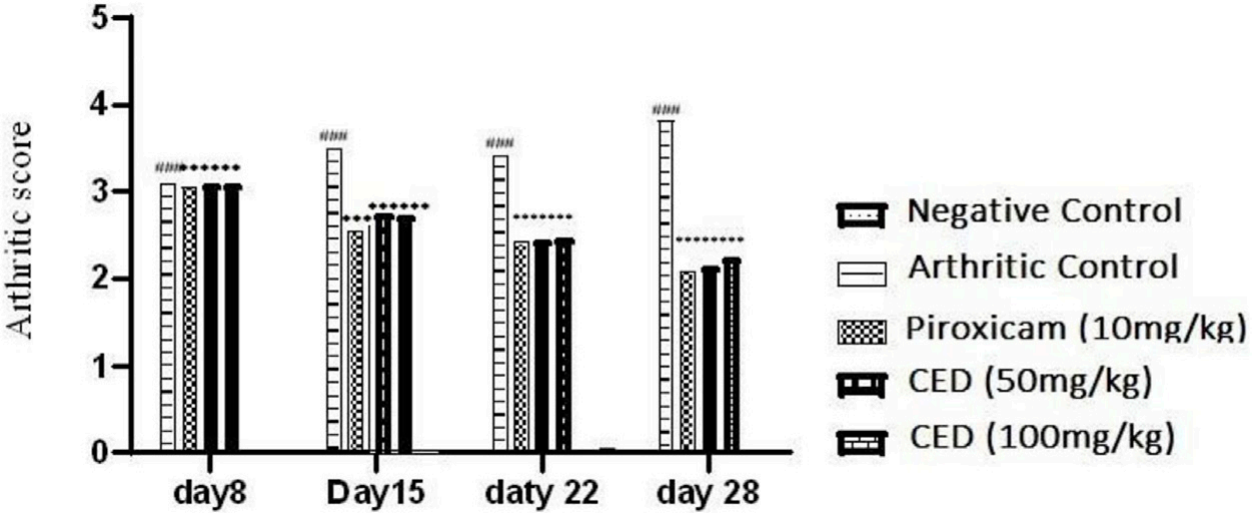
CED reduced Arthritic more In KA-induced arthritic model.

In assessing nociceptive behavior, various parameters were evaluated to monitor the progression of arthritis. Paw volume and arthritis score were measured on days 0, 8^th^,15^th^, 22^nd^ and 28^th^ day after arthritis induction Figure 1. The thickness of the right hind paw was quantified using a calliper. The severity of arthritis, represented as the CED score, was categorized as follows: 0 indicated an insignificant change, 1 represented slight erythema or bulging of digits, 2 denoted medium swelling and erythema, 3 indicated high swelling and erythema, including the ankle, and 4 represented ankylosis and an inability to bend the ankle (Kumar et al., 2006).

To assess thermal hyperalgesia, a hot plate maintained at 55°C was used. The latency to the first sign of licking the paw or jumping was recorded to determine the nociceptive threshold to heat. Mechanical allodynia was evaluated using von Frey filaments. The rats were placed on an elevated metal wire floor in a 30 × 30 × 30 cm box, and the withdrawal threshold of the paw was determined using a series of von Frey filaments with varying forces. A significant response was considered when the rat responded to three out of five successive trials, indicating the presence of mechanical allodynia Table 1.

**TABLE 1:** CED attenuated arthritic score.

Days	Vehicle control	Positive control	Piroxicam (10 mg\kg)	CED (50 mg\kg)	CED (100 mg\kg)
AS at day 8	0.000 ± 0.000	3.087 ± 0.0067	3.058 ± 0.0064	3.079 ± 0.00342	3.085 ± 0.00477
AS at day 15	0.000 ± 0.000	3.488 ± 0.0224	2.547 ± 0.00852**	2.783 ± 0.0077**	2.755 ± 0.0077**
AS at day 22	0.000 ± 0.000	3.418 ± 0.0015	2.417 ± 0.00428**	2.484 ± 0.008**	2.509 ± 0.00542**
AS at day 28	0.000 ± 0.000	3.818 ± 0.0076	2.088 ± 0.00174**	2.129 ± 0.0107**	2.233 ± 0.0107**

### 2.4 Biochemical assays

For the measurement of cytokines, the serum levels of interleukin 1 β (IL-1β) and tumor necrosis factor α (TNF-α) were measured using Prostaglandin E2 (PG-E2) ELISA Kit (CSB-E07967r) following the manufacturer’s instructions (Nair et al., 2010). The measurement of oxidative stress markers included the levels of malondialdehyde (MDA), an end product of lipid peroxidation, and superoxide dismutase (SOD) activity were assessed in the serum to determine oxidative stress. Additionally, thiol concentration and glutathione peroxidase (GPX) activity were measured using appropriate assays.

### 2.5 Statistical analysis

The data were analysed using GraphPad Prism version 6.0. Descriptive statistics were used to present the data as mean ± SEM (Standard Error of the Mean). Behavioural parameters were analysed using a two-way analysis of variance (ANOVA) followed by tacchi test Bonferroni’s test for *post hoc* comparisons. Biochemical factors were analysed using one-way ANOVA with *p < 0.05* considered statistically significant.

### 2.6 Ethical considerations

All experiments followed the guidelines and regulations for the care and use of laboratory animals. The study protocol was approved by the University of Lahore, Institutional Research Ethics Committee (IREC) before the initiation of the experiments via Trial Registry No: IREC-PHM-21-00565.

## 3 Results

### 3.1 Therapeutic effects of CED derivative on paw edema in FCA-induced arthritic rat model

**FIGURE 2:**
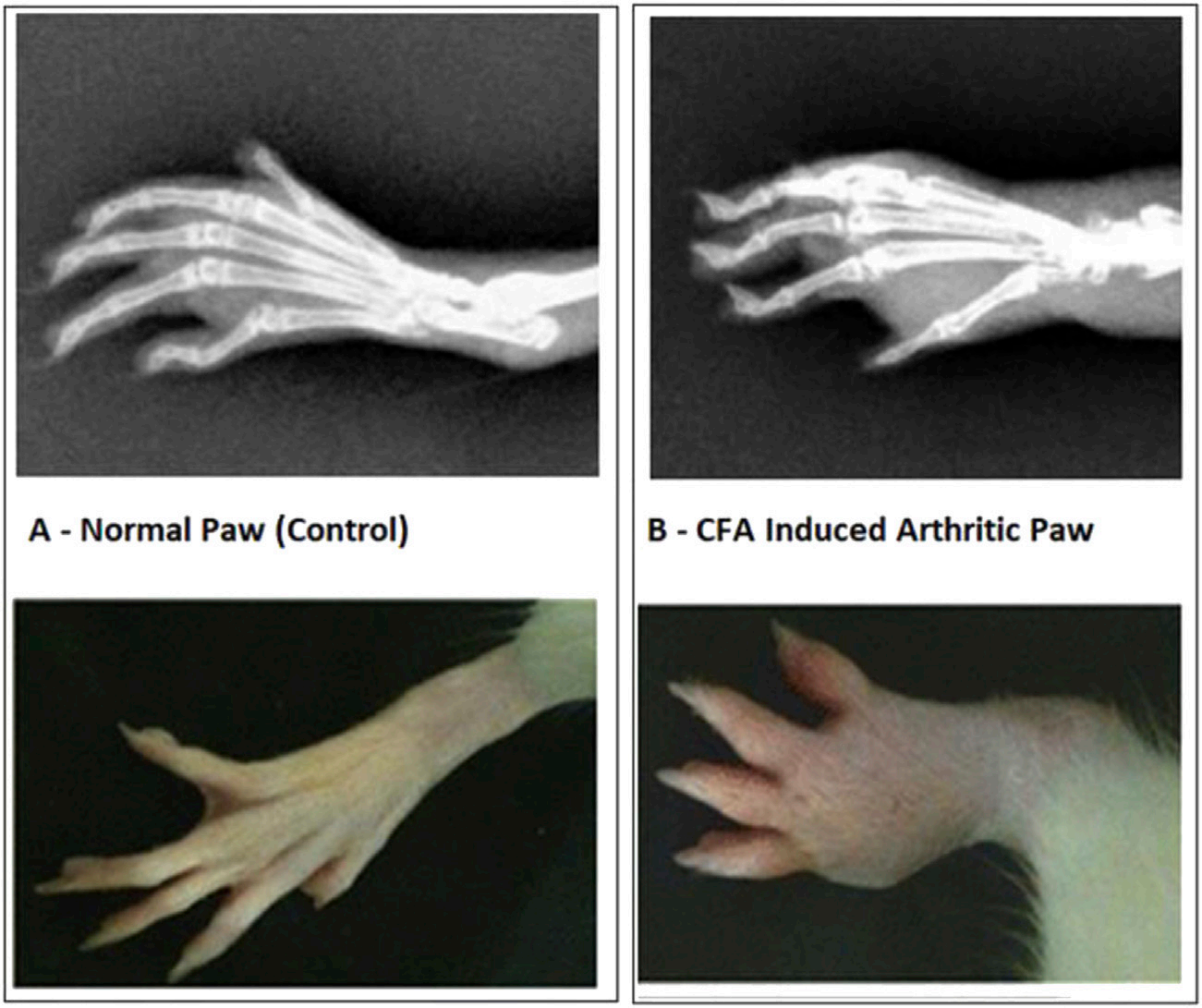
**(A)** Normal Paw and CFA Induced Arthritic Paw. Paw oedema over the period of 28 days. The significant increase in rat paw edema was statistically significant (*p* < 0.001) compared to the control group of rats with arthritis. Notably, when monitoring paw edema in the piroxicam-treated group at various intervals with a gap of 6 days, CED demonstrated significant inhibitory effects (*p* < 0.001) compared to the arthritic group in each 6-day interval throughout the 28-day experiment.

**FIGURE 3:**
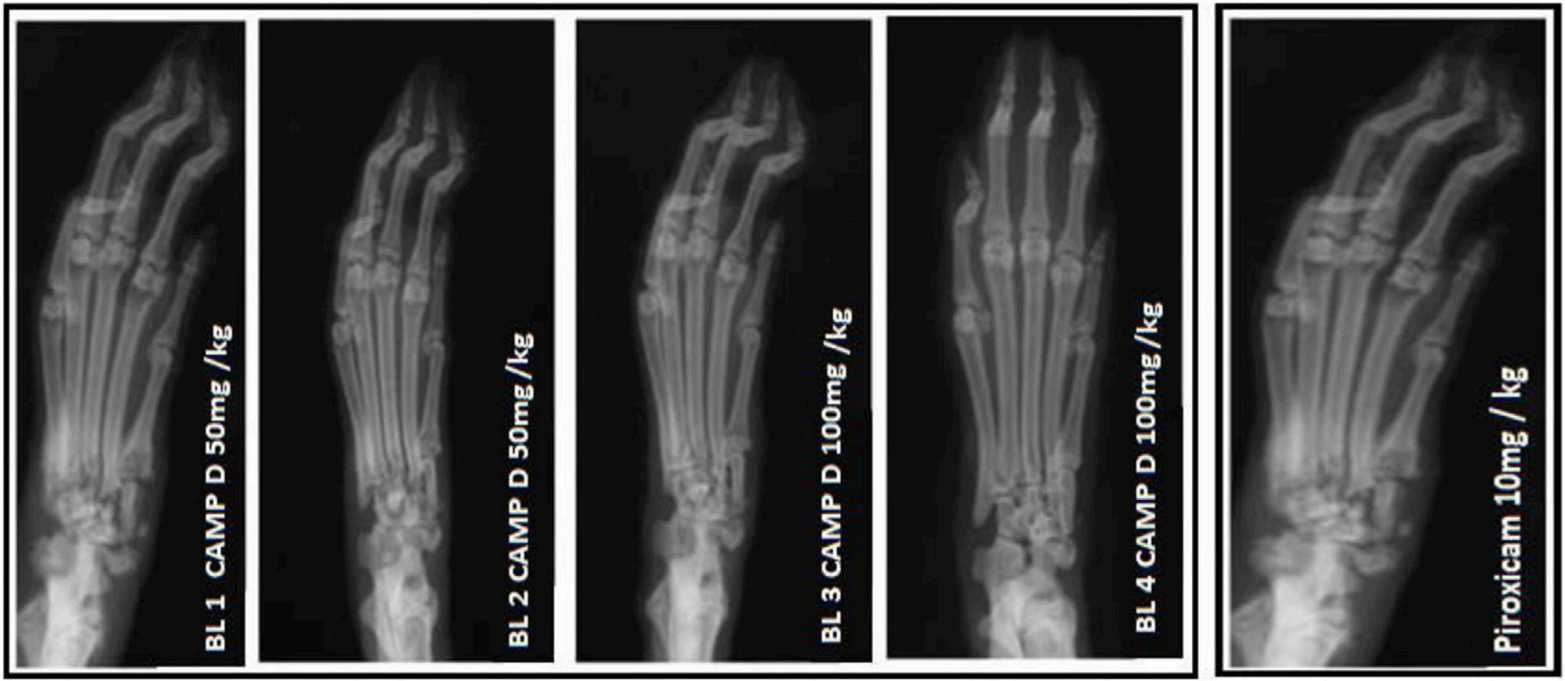
X-ray photographs of the normal rat paw illustrating the AP view of X-rays.

In the FCA-induced Arthritic rat model, CED (Compound E Derivative) was administered and compared to the illness group. The results revealed significant findings (***p < 0.001), as depicted in Figure 2. Over 28 days, CED exhibited a gradual increase in paw edema levels. At day 28, when compared to the positive control group (1.52 ± 0.05), both the CED derivative at 50 mg/kg (0.69 ± 0.02) and 100 mg/kg (0.79 ± 0.017), as well as piroxicam (0.74 ± 0.003), demonstrated inhibitory effects on paw edema. The paw edema progression was assessed, and the results were analyzed by the end of the 22nd day. On the 28th day, the difference in paw edema inhibition between the CFA-induced and positive control groups was 1.52 ± 0.05. X-rays of rats’ lower limbs were taken using a Siemens X-ray machine to assess joint variations. The analysis was based on joint space and swollen soft tissue. Figure 3 illustrates anteroposterior (AP) view of X-rays of rat paws on the 21st and 28th days for both the normal and arthritic rat groups. X-rays were also utilized to evaluate arthritis using piroxicam 10 mg/kg. X-ray photographs of the normal rat paw showed the presence of 8 Joint Spaces, 8 Soft tissues, and 3 metatarsal bones. On the other hand, CFA rats from the vehicle treatment group exhibited several deformities, including significant paw edema, osteophyte development, decreased joint spacing, and bone degradation.

The results in Table 2 provided evidence of the influence of CED on the decrease in paw edema levels at different time points (day 8, day 15, day 22, and day 28). The data indicated a statistically significant decrease in paw edema when treated with CED, with a *p-value* of 0.001, highlighting the potential therapeutic effects of the compound. Table 2 presents the results of the paw edema measurements as an influence of campesterol ester derivatives treatment at different time points (day 8, day 15, day 22, and day 28) in the FCA-induced arthritic rat model. The values are represented as mean ± standard error of the mean (SEM). The negative control group represents the untreated normal rats, while the arthritic control group represents rats with arthritis who did not receive any treatment. The positive control group was treated with piroxicam (10 mg/kg), a known anti-inflammatory drug for arthritis management. The two treatment groups received CED derivatives at 50 mg/kg and 100 mg/kg, respectively.

**TABLE 2:** CED significantly reduced paw edema in FCA induced arthritis model of different concentrations.

Days	Negative control	Arthritic control	Piroxicam (10 mg/kg)	CED (50 mg/kg)	CED (100 mg/kg)
PE at day 8	0.4683 ± 0.0060	0.9775 ± 0.00427	0.9843 ± 0.0039	0.9618 ± 0.004549	0.9620 ± 0.00432
PE at day 15	0.4767 ± 0.000	1.172 ± 0.0069	0.8548 ± 0.0070***	0.8707 ± 0.00584***	0.8748 ± 0.00302***
PE at day 22	0.4000 ± 0.0131	1.373 ± 0.00971	0.8427 ± 0.01710***	0.8747 ± 0.00624***	0.8873 ± 0.0118***
PE at day 28	0.4517 ± 0.01682	1.473 ± 0.00461	0.7240 ± 0.003055***	0.6877 ± 0.00224***	0.7848 ± 0.00137***

During the experiment conducted over the course of 28 days, the effects of various treatments on paw edema levels were investigated. On Day 8, the negative control group exhibited a paw edema level of 0.49 ± 0.006, while the arthritic control group showed a significantly higher level of 0.99 ± 0.004. The treatment groups administered with Piroxicam (10 mg/kg), CED derivative (50 mg/kg), and CED derivative (100 mg/kg), displayed paw edema levels of 0.89 ± 0.003, 0.98 ± 0.005, and 0.97 ± 0.004, respectively.

By Day 15, the negative control group showed a paw edema level of 0.46 ± 0.001, while the arthritic control group exhibited a substantially higher level of 1.18 ± 0.006. The Piroxicam-treated group showed a considerable reduction in paw edema to 0.85 ± 0.007 (***p < 0.001), and the CED derivative (50 mg/kg) and CED derivative (100 mg/kg) treated groups also displayed significant reductions to 0.88 ± 0.005 (***p < 0.001) and 0.87 ± 0.003 (***p < 0.001), respectively.

On Day 22, the negative control group showed a similar paw edema level of 0.45 ± 0.01, while the arthritic control group displayed a higher level of 1.38 ± 0.009. The Piroxicam-treated group exhibited a considerable reduction in paw edema to 0.84 ± 0.01 (***p < 0.001), and the CED derivative (50 mg/kg) and CED derivative (100 mg/kg) treated groups also showed substantial reductions to 0.87 ± 0.006 (***p < 0.001) and 0.88 ± 0.01 (***p < 0.001), respectively.

Finally, on Day 28, the negative control group displayed a paw edema level of 0.44 ± 0.01, whereas the arthritic control group had a significantly higher level of 1.48 ± 0.004. The Piroxicam-treated group showed a considerable reduction in paw edema to 0.72 ± 0.003 (***p < 0.001), while the CED derivative (50 mg/kg) treated group exhibited a significantly reduced level of 0.68 ± 0.002 (***p < 0.001). Similarly, the CED derivative (100 mg/kg) treated group displayed a considerable reduction in paw edema to 0.78 ± 0.001 (***p < 0.001). These findings indicate the potential anti-inflammatory effects of the CED derivative treatments in reducing paw edema levels in the arthritic model, comparable to or even better than the standard Piroxicam treatment.

**FIGURE 4:**
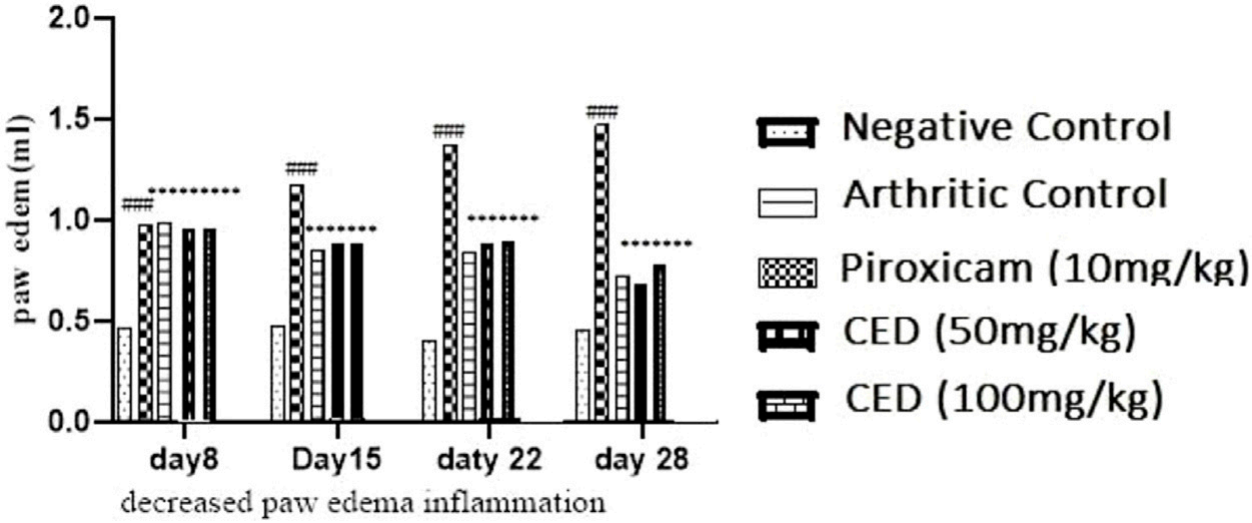
The reduction of paw edema (inflammation] seen at different day intervals *p* < 0.001 in contrast with arthritic control group.

The results demonstrate that the CED derivative at both 50 mg/kg and 100 mg/kg doses effectively reduced paw edema levels compared to the arthritic control group. The inhibitory effects were statistically significant and comparable to the positive control group treated with piroxicam. These findings suggest the potential therapeutic efficacy of the CED derivative in mitigating paw edema associated with arthritis in the rat model Figure 4.

### 3.2 Histopathological parameters

**FIGURE 5:**
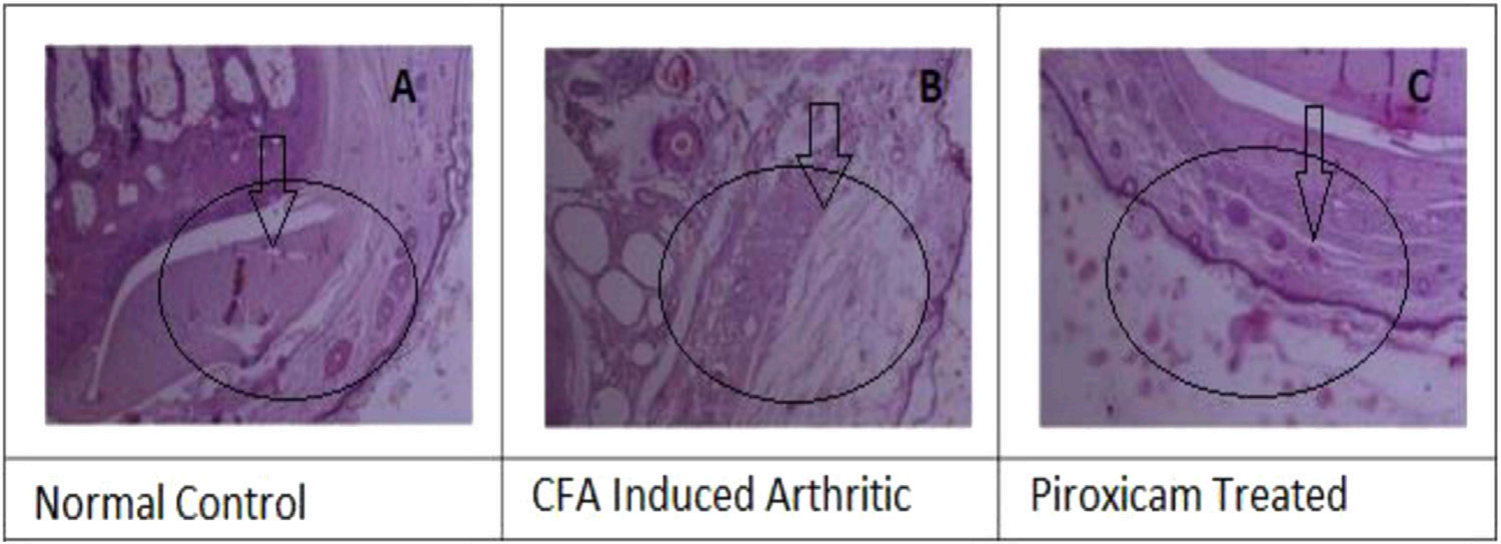
**(A)** Histopathology, of normal cells, **(B)** Histopathology of the diseased paw of rats, and **(C)** Hislopathology of Piroxicarn-treatod.

The histopathology analysis was conducted on rat paw samples to study the effects of different treatments. The normal paw histopathology showed (Figure 5A) a typical healthy synovial membrane layer surrounded by concave synovial lining epithelium (Figure 5B). There was no evidence of inflammatory cell influx or abnormal tissue formations, such as pannus, were present. The normal paw histopathology showed a typical healthy synovial membrane layer surrounded by concave synovial lining epithelium. There was no evidence of inflammatory cell influx or abnormal tissue formations, such as pannus, were present. The paw was treated with piroxicam Figure 5C showed inflammation in the synovial membrane, but it was localized and characterized by clumps of inflammatory cells. Infamed-pauci regions were also present, and a substantial plaque of fibrovascular tissue was observed.

**FIGURE 6:**
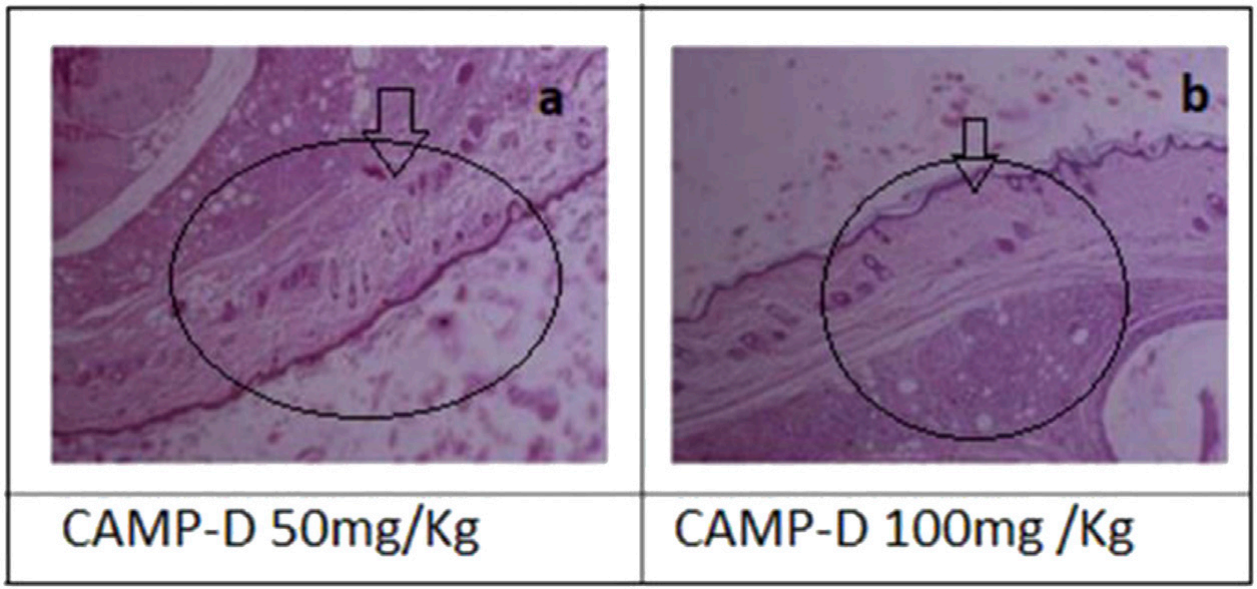
Histopathnlogy **(A)** of CED (50 mg/kg) treated rat paw and **(B)** Histopathology of rat Paw treated with CED 100 mg/kg.

The results showed that CED attenuated various histopathological parameters associated with inflammation and arthritis. The examination of histopathological parameters revealed that CED compounds led to a reduction in inflammation in the rats compared to the control group (value: 2.6 ± 0.01), the rats treated with CED at a dose of 50 mg/kg showed reduced inflammation (value: 1.81 ± 0.004), as shown in Figure 6A. Similarly, the rats treated with CED at a dose of (Figure 6B) 100 mg/kg also exhibited reduced inflammation (value: 1.86 ± 0.005), indicating the significant anti-inflammatory effects of CED.

In the paw treated with CED at a dose of 50 mg/kg, inflammatory cells were in the synovial membrane. Additionally, several regions of inflamed pauci were identified. The inflammatory cells seemed to surround the synovial membrane in a concerted manner. In the paw treated with CED at a higher dose of 100 mg/kg, inflammatory cells were scattered around the synovial membrane. Most of the pauci-inflamed regions were identified within the synovial membranes. A detailed examination revealed no significant focus on pannus production.

### 3.3 Anti-inflammatory effects of CAMP derivatives on inflammatory cells, pannus formation and bone erosion in arthritis

**FIGURE 7:**
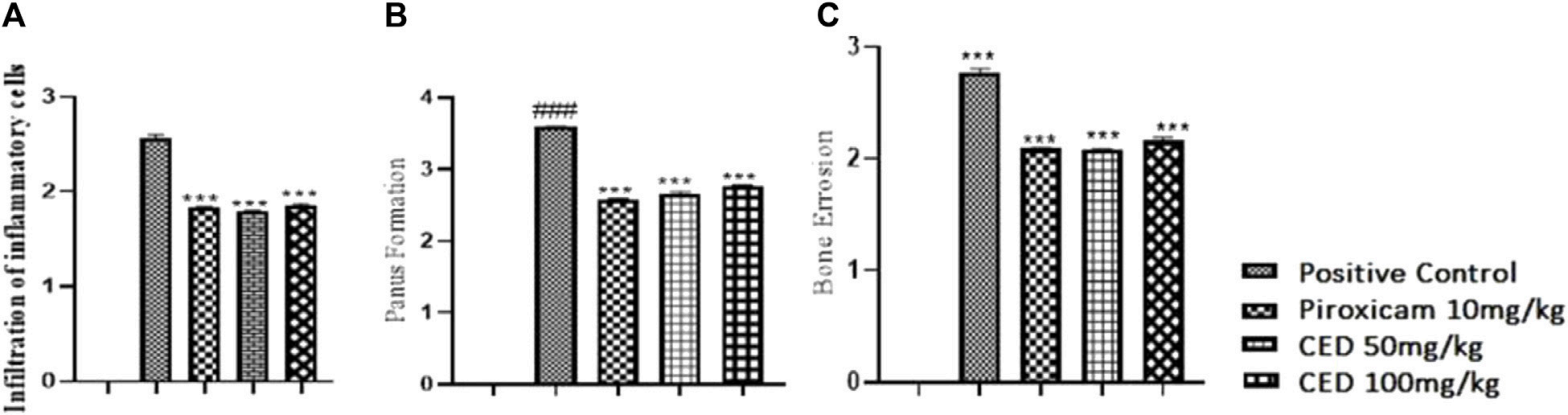
Shows **(A)** Reduction in infiltarion of inflammatory cells, **(B)** pannus formation, and **(C)** bone erosion Histopathological analysis demonstrated the impact of CED compared to the positive control and standard drug (piroxicam). The vehicle control group had negligible values for inflammation (HP INF). Pannus formation (HP PF). And bone erosion (HP BE). The positive control group had significantly higher values for these parameters. Piroxicam at 10 mg/kg reduced these values, and CED at doses of 50 mg/kg and 100 mg/kg exhibited even lower values for all parameters.

Treatment with CAMP derivatives (CED) showed significant anti-inflammatory effects with CED at doses 50 mg\kg and 100 mg\kg in an experimental rat model of arthritis. CED at a dose of 100 mg/kg significantly reduced pannus formation (*p* < 0.001) and bone erosion compared to the arthritic control group (Figure 7). The symbol ### indicates comparison between arthritic and control groups, while *** shows treatment vs. arthritic control group. There is no plotting of the bar graph of the normal group due to taking of value as 0.

Figure 7 shows a reduction in infiltration of inflammatory cells, pannus formation, and bone erosion Histopathological analysis demonstrated the impact of CED compared to the positive control and standard drug (piroxicam). The vehicle control group had negligible values for inflammation (HP INF), pannus formation (HP PF), and bone erosion (HP BE). The positive control group had significantly higher values for these parameters. Piroxicam at 10 mg/kg reduced these values, and CED at doses of 50 mg/kg and 100 mg/kg exhibited even lower values for all parameters (Figure 7).

In the normal vehicle group, no pannus formation, cartilage erosion, ulceration erosion, or infiltration of mononuclear inflammatory cells was observed. The disease group displayed pannus formation, cartilage erosion, ulceration erosion, and infiltration of mononuclear inflammatory cells. Piroxicam treatment showed no pannus formation but mild cartilage erosion. No ulceration erosion or infiltrations of mononuclear inflammatory cells were observed in the piroxicam group, as well as in the CED (50 mg/kg) and CED (100 mg/kg) groups.

Overall, CED treatment at both 50 mg/kg and 100 mg/kg doses effectively reduced pannus formation, cartilage erosion, ulceration erosion, and infiltration of mononuclear inflammatory cells, similar to the standard treatment with piroxicam. These promising results suggest that CED could be a potential treatment option for arthritis.

### 3.4 Histopathological interpretation of liver

**FIGURE 8:**
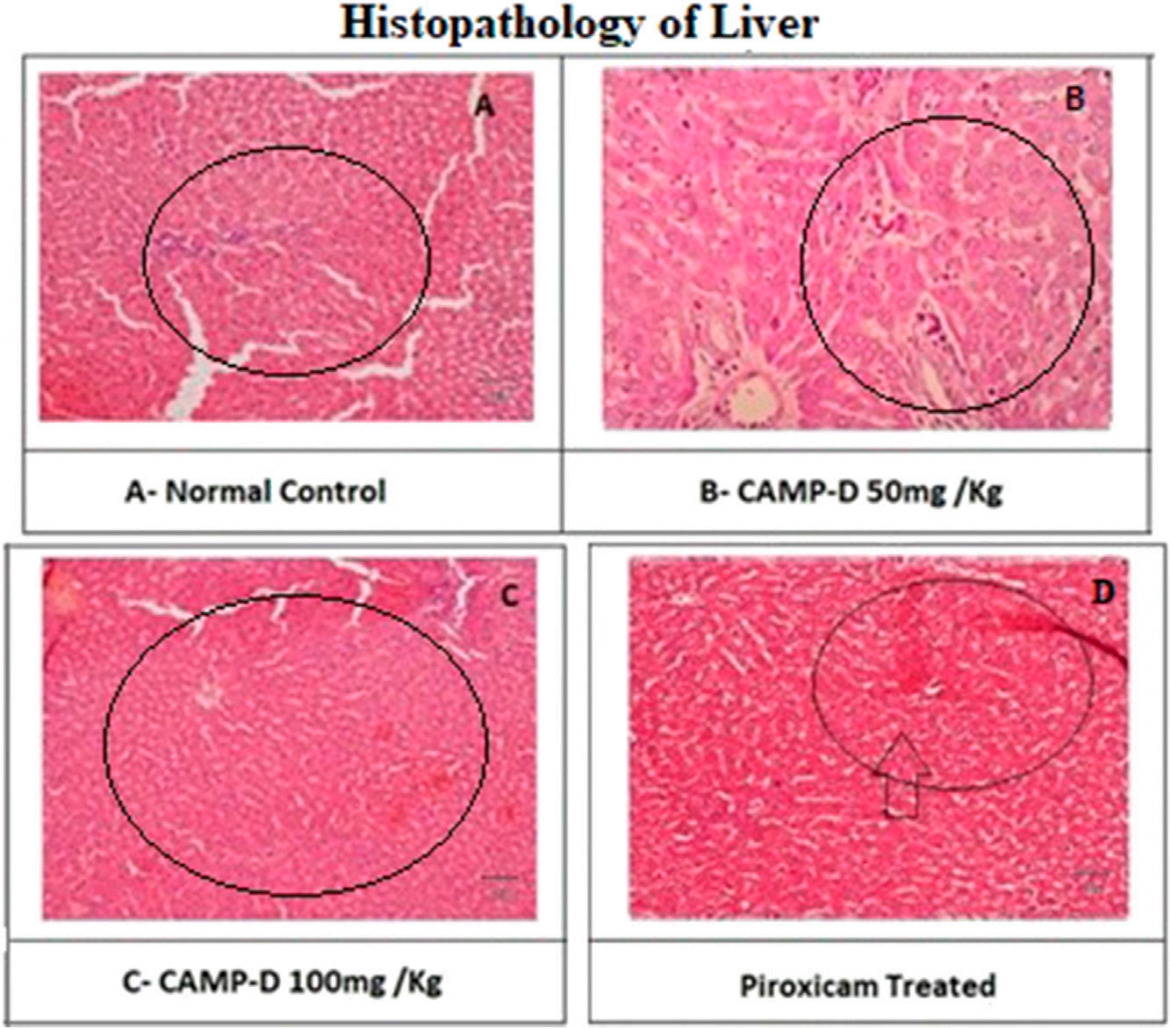
Histopathology of rat Liver treated. With Piroxicam. **(A)** Normal liver. **(B)** CED50mg\kg. **(C)** CAMP 100mg\kg. **(D)** Piroxicam.

The normal liver in rats appeared healthy and showed no signs of inflammation, edema, erosion, ulcer, or fibrosis. This is considered as the control group (vehicle control). In contrast, the liver of rats induced with CFA (Complete Freund’s Adjuvant) showed moderate cellular swelling in the hepatocytes, indicative of liver damage due to inflammation, as shown in Figure 8. The liver sections from rats treated with CED (Curcumin-loaded Elastic Vesicles) at doses of 50 mg/kg and 100 mg/kg showed no signs of inflammation, edema, erosion, ulcers, or fibrosis, similar to the control group. This suggests that CED treatment did not cause any observable liver damage or adverse effects Table 3. On the other hand, the liver sections from rats treated with standard piroxicam (a nonsteroidal anti-inflammatory drug) showed mild inflammation, edema, erosion, ulcers, or fibrosis, indicating some degree of liver injury due to piroxicam treatment.

**TABLE 3:** Histopathological interpretation of liver.

Histopathological changes	Normal vehicle group	Disease group	Standard group	CED 50 mg/kg	CED 100 mg/kg
Sinusoidal congestion	None	Significant	Mild	Significant	Significant
Cellular Inflammation	None	Significant	Mild	None	None
Tissue changes	None	Significant	None	None	None
Infiltration of inflammatory cells	None	Significant	Mild	Mild	Mild

### 3.5 Histopathological interpretation of kidneys

**FIGURE 9:**
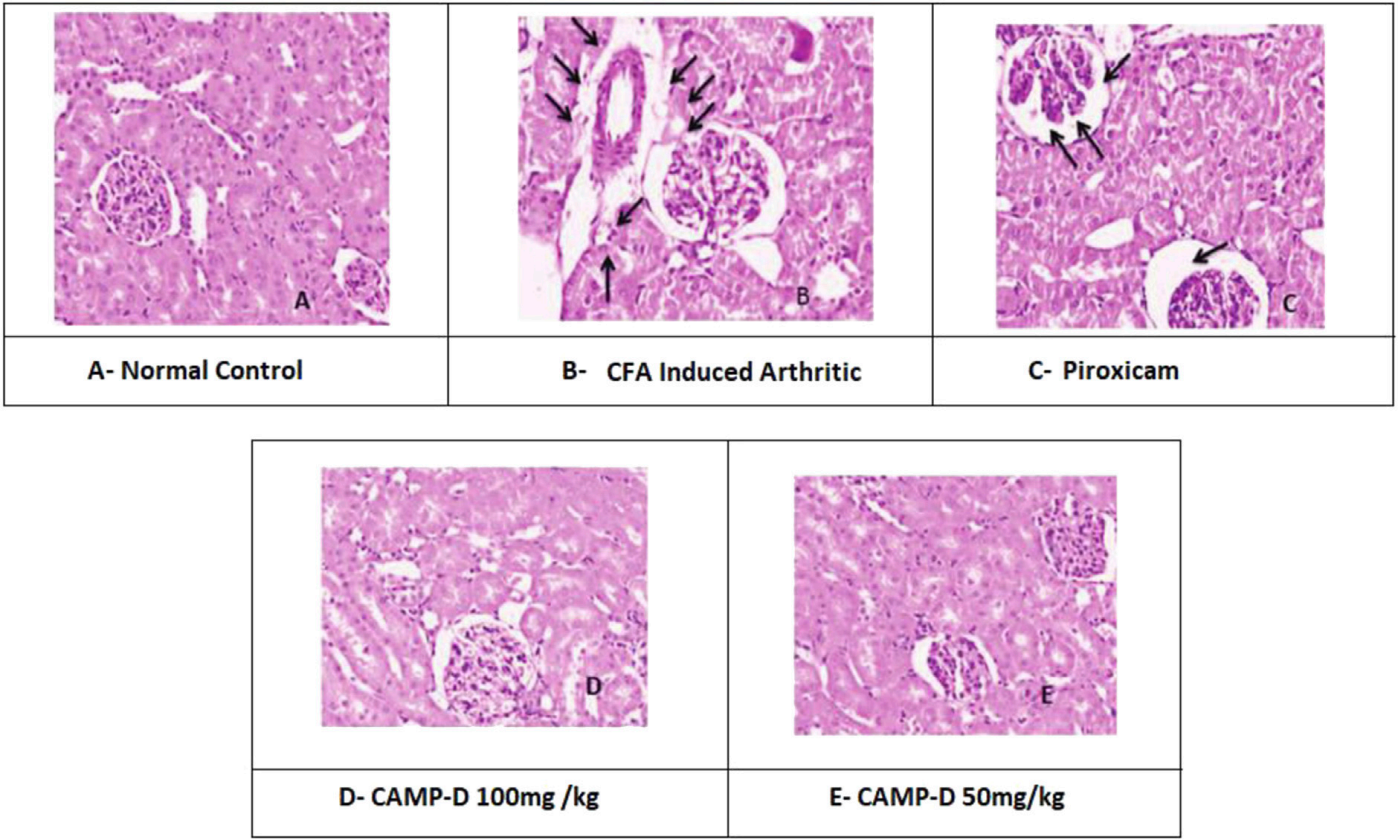
Histopathogical interpretation of Kidney treated **(A)** Normal kidney, **(B)** CFA-induced Kidney, **(C)** Piroxicam-treated kidney, **(D)** CAMP-D 100mg\kg, **(E)** CAMP-D 50mg\kg.

The histopathological analysis of normal kidneys from the control group (vehicle control) showed a regular and healthy kidney structure. However, the kidneys of rats induced with CFA displayed distortions in the usual kidney structure, indicating kidney damage due to inflammation, as shown in Figure 9 the treatment with piroxicam and CED at 50 mg/kg and 100 mg/kg doses appeared to protect the kidneys from inflammation-induced damage. Post-treatment histopathology of kidneys with piroxicam showed protective variations in the renal structures, such as tubules and glomeruli, in a dose-dependent manner. Similarly, in the CED 100 mg/kg treated group, the shielding modifications were evident in the morphology of tubular epithelial cells and renal corpuscle/parietal epithelium. Additionally, the CED 50 mg/kg treated group (Table 4) showed kidney histology closely resembling the control group, indicating minimal kidney damage and protection from inflammation.

**TABLE 4:** Histopathological changes in tissue structures, cellular swelling, coagulative necrosis, and inflammation in different treatment groups.

Histopathological changes	Normal vehicle group	Disease group	Standard group	CED 50 mg\kg	CED 100 mg\kg
Tissue structures change	None	Tissue damage and necrosis found	Mild	None	None
Cellular swelling	None	Significant	Mild swelling	None	None
Coagulative necrosis	None	Significant necrosis	Mild necrosis	None	None
Inflammation and infiltration	No infiltration found	Inflammatory cells infiltrated into renal tubular epithelial cells	Mild Infiltration	None	None

### 3.6 CED treatment on mRNA expression levels of inflammatory markers

**FIGURE 10:**
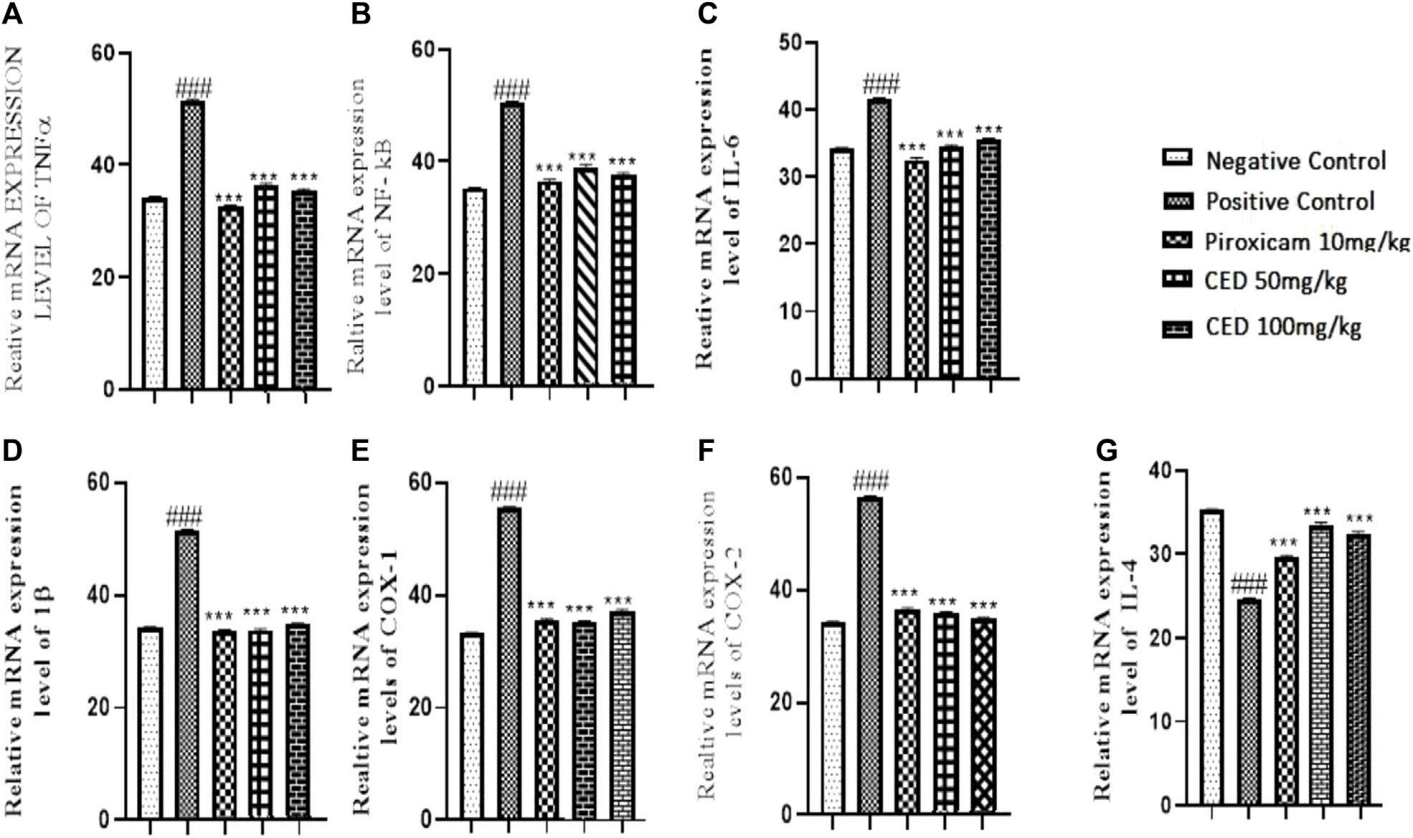
mRNA expression levels of inflammatory markers in different treatment groups, The graph reveals CED decreases. The mRNA expression levels of **(A)** TNF‐α.: **(B)** NF‐kE **(C)** I L‐6, **(D)** I by **(E)** COX‐I, **(F)** COX‐II, while increses the 1L‐4 **(G)** at different day intervals.P < OA:11 in contrast with arthritic control group.

The effects of CED compounds on mRNA expression levels of pro-inflammatory and anti-inflammatory markers were evaluated in an arthritic rat model, and the results were compared with a positive control group treated with piroxicam and a vehicle control group. The findings demonstrated the downregulation of pro-inflammatory markers TNF-α, IL-1ß, IL-6, NF-KB, MMP3, COX-I, COX-II and upregulation of anti-inflammatory markers IL-4 following CED treatment. TNF-α, a pro-inflammatory cytokine, showed significantly reduced mRNA expression levels in both CED treatment groups (50 mg/kg and 100 mg/kg) compared to the vehicle control and piroxicam-treated groups (*p* < 0.001). The mean values for TNF-α were (33.53 ± 0.12) for the vehicle control (37.47 ± 0.11) for CED 50 mg/kg, and (36.45 ± 0.11) for CED 100 mg/kg. The graph (Figure 10A) visually depicted the downregulation of TNF-α mRNA expression.

Similarly, the expression levels of NF-κB, a transcription factor involved in inflammatory responses, were significantly reduced by CED treatment. In the piroxicam-treated group, NF-κB expression was (37.53 ± 0.12), while CED 50 mg/kg showed (39.63 ± 0.23) and CED 100 mg/kg showed (38.45 ± 0.18). The reduction in NF-κB expression levels was evident in the treated groups compared to the vehicle control and was illustrated in the graph (Figure 10B).

Moreover, CED treatment resulted in a significant downregulation of other inflammatory markers. The mRNA expression levels of IL-6 were decreased (Figure 10C) in both CED treatment groups (50 mg/kg and 100 mg/kg) in comparison to the vehicle control and piroxicam-treated groups. The mean values for IL-6 were (34.47 ± 0.11) for CED 50 mg/kg and (35.47 ± 0.10) for CED 100 mg/kg, indicating reduced inflammatory activity. In contrast, the piroxicam-treated group showed a mean value of (32.53 ± 0.12).

Furthermore, the mRNA expression levels of IL-1ß, COX-1 and COX-2 were significantly downregulated by CED treatment (Figures 10D–F). In the piroxicam-treated group, COX-1 expression was (36.53 ± 0.12), whereas CED 50 mg/kg showed (35.47 ± 0.11) and CED 100 mg/kg showed (36.47 ± 0.10). Similarly, for COX-2, the piroxicam-treated group had a mean value of (35.53 ± 0.12), and the CED treatment groups showed (36.47 ± 0.11) for 50 mg/kg and (35.47 ± 0.12) for 100 mg/kg. Additionally, the expression levels of IL-4 were also significantly upregulated in the Piroxicam and CED treatment groups compared to the arthritic control group (Figure 10G). For IL-4, the piroxicam-treated group exhibited a mean value of (29.53 ± 0.12), while CED 50 mg/kg and CED 100 mg/kg showed (33.47 ± 0.11) and (32.42 ± 0.09), respectively.

In conclusion, the results from this study indicated that CED compounds effectively downregulated the mRNA expression levels of pro-inflammatory markers, such as TNF-α, NF-κB, IL-6, COX-I, COX-II and IL-1ß, while also upregulating some anti-inflammatory markers like IL4 that are shown in (Figure 10G) In the treatment group as compared to positive control arthritic group. These findings suggest the potential of CED as a promising anti-inflammatory agent warranting further investigation. The sign of ### shows a comparison between the arthritic control group and the vehicle group in Figures. A, B, C, D, E and F). While ***shows comparision between the treatment group and the arthritic control group.

### 3.7 Hematological and biochemical parameters in arthritic rats

**FIGURE 11:**
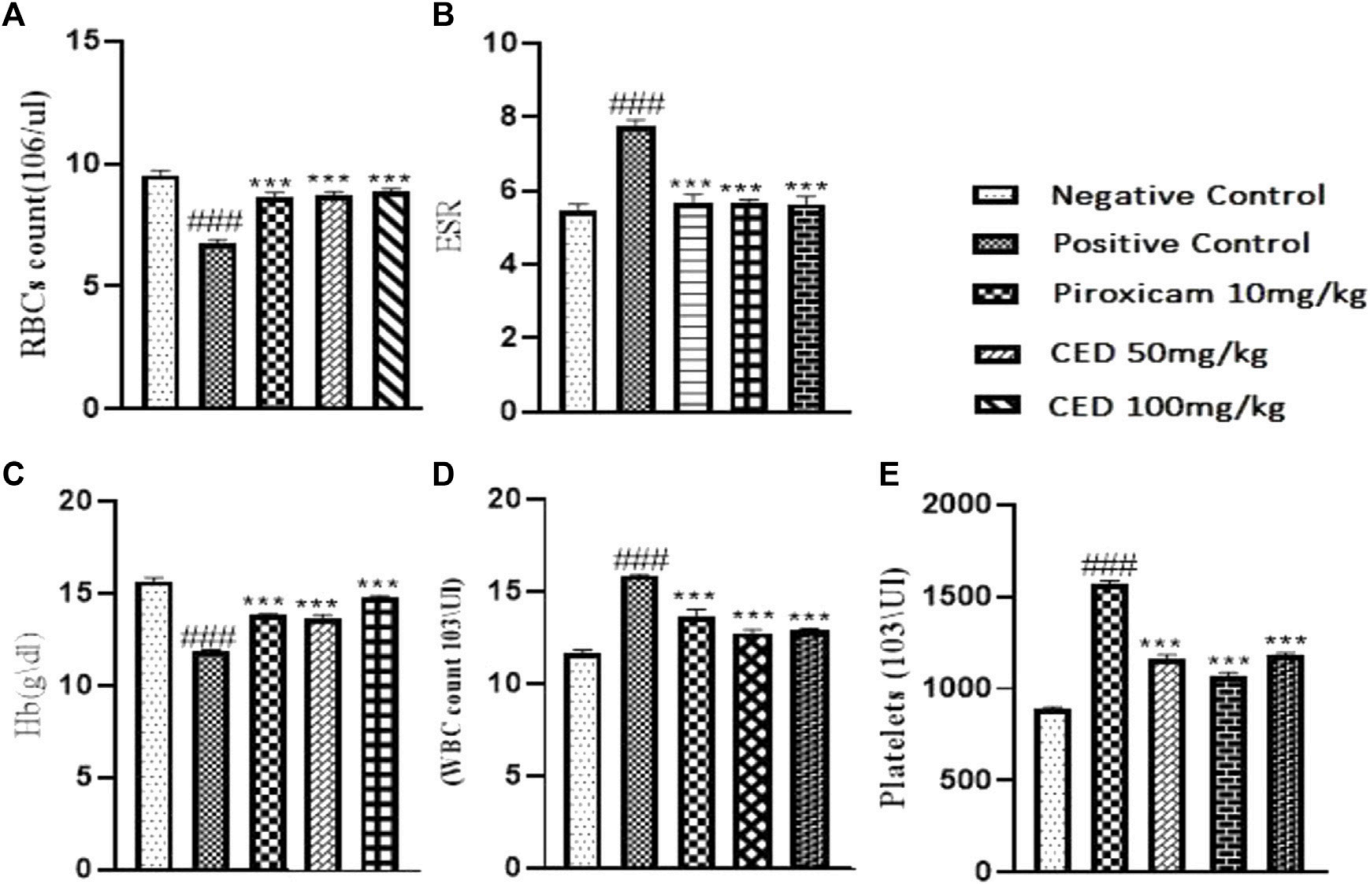
Shows the summarized impact of CED doses tin Haemotological parameters on arthritic rats.

**FIGURE 12:**
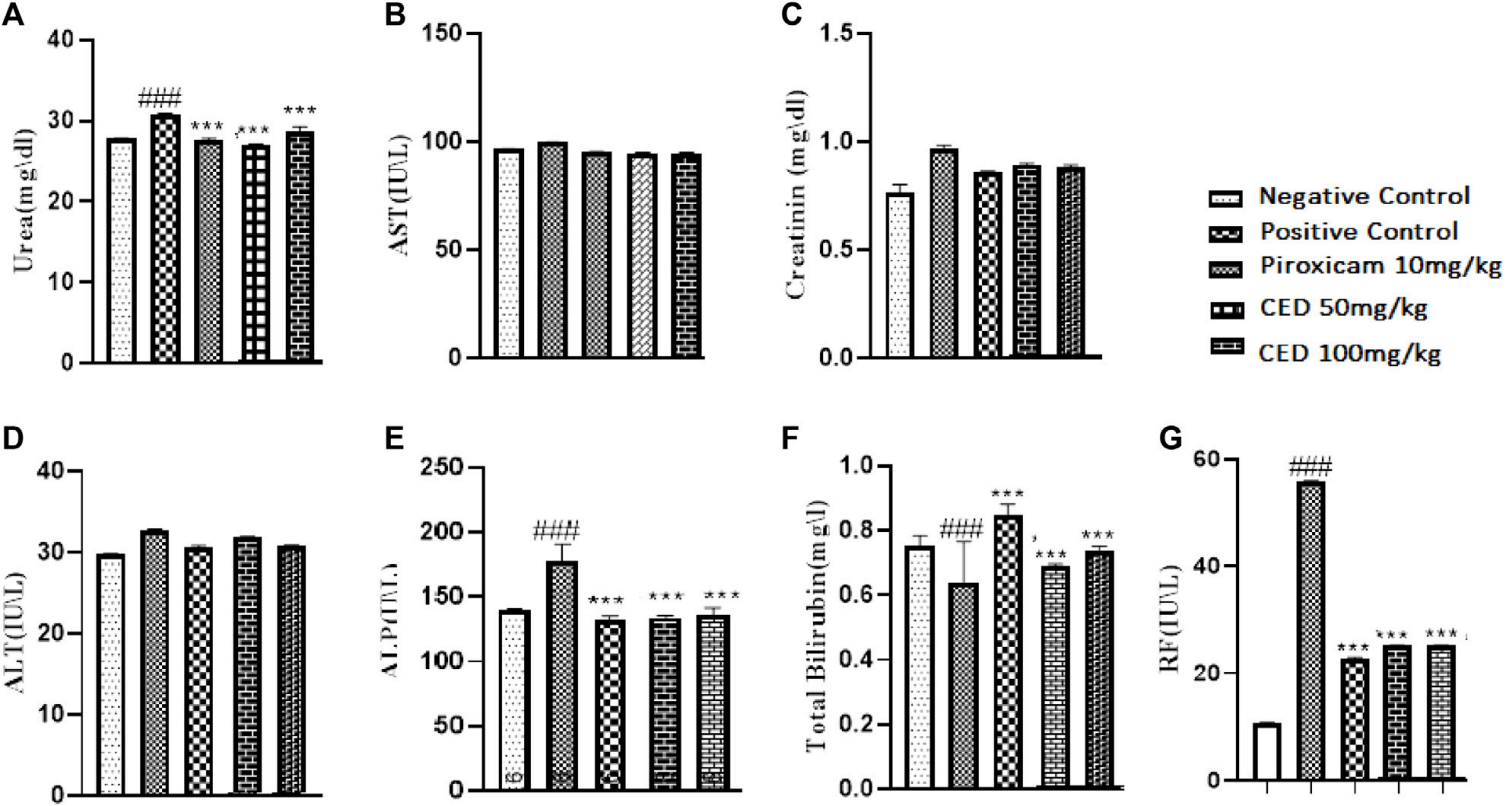
Shows the summarized impact of CED doses on Biochemical parameters on arthritic rats.

The treatment with CED affected hematological parameters in the arthritic rats. The arthritic control group showed a significant reduction in red blood cell (RBC) count, an increase in erythrocyte sedimentation rate (ESR), and a decrease in hemoglobin (Hb) content compared to the vehicle control group Figure 11. However, treatment with piroxicam and CED at 50 mg/kg and 100 mg/kg doses resulted in significant improvements in these hematological parameters. The RBC count increased, ESR levels decreased, and Hb content increased, indicating a positive effect of CED on the blood parameters in arthritic rats. CED treatment also normalized the platelet count compared to the arthritic control group. Moreover, CED treatment also affected biochemical parameters in the arthritic rats (Figure 12).The levels of urea, creatinine, total bilirubin, aspartate aminotransferase (AST), alanine aminotransferase (ALT), and alkaline phosphatase (ALP) were all brought closer to normal levels by CED treatment at both 50 mg/kg and 100 mg/kg doses. This indicates that CED positively impacted kidney and liver function, as these biochemical parameters indicate kidney and liver health. The levels of rheumatoid factor, which indicates autoimmune activity, were also significantly reduced by CED treatment. The sign denoted by### shows comparision between the arthritic group and the vehicle control group while*** shows comparision between the treatment group and the arthritic control group.

## 4 Discussion

The experimental model of Campesterol Derivatives exhibits several human rheumatoid arthritis (RA) aspects, such as hypersensitivity to mechanical and heat stimuli, polyarticular irritation, and joint structure deterioration (Nazir et al., 2023).This model is widely used to study RA pathogenesis and assess potential therapeutic targets for treating RA (Martínez-Hernández et al., 2020). Intraplantar injection of Campesterol Derivatives induces a strong immune response in rat paws mediated by T-lymphocytes, which interact with dendritic cells, macrophages, and monocytes, leading to the production of proinflammatory cytokines like TNF-α, IL-1β, and IL-6 in the synovial membrane. The interaction among these proinflammatory mediators results in synovial inflammation and cartilage or bone destruction, making TNF-α blockade an effective treatment for reducing RA symptoms. IL-1β and TNF-α are also recognized as therapeutic targets for RA treatment due to their role in chronic inflammatory pain associated with RA (Sartinah et al., 2022).

Previous studies have shown that sesquiterpenes possess anti-inflammatory, analgesic, and antioxidant properties (Berlin Grace and Monisha, 2022). In this study, we investigated the potential protective effects of Campesterol Derivatives against Campesterol Derivative-induced arthritis in rats. The results demonstrated that Campesterol Derivatives improved nociception behavior and exhibited anti-inflammatory and antioxidant effects by reducing the thickness of rat paws and inhibiting the release of inflammatory mediators IL-1β and TNF-α. Similarly, Piroxicam, a nonsteroidal anti-inflammatory drug, reduced paw thickness, showed antinociceptive behavior, and decreased serum levels of TNF-α and IL-1β. Antioxidant therapy is known to be beneficial in oxidative stress and inflammation-related diseases, as an imbalance between prooxidants and antioxidants leads to the excessive production of reactive oxygen species (ROS) (Giménez-Bastida et al., 2021).

ROS play a crucial role in arthritis pathogenesis, as they directly or indirectly activate latent collagenases, damaging the matrix components and contributing to joint inflammation. Mitochondrial ROS stimulate the production of proinflammatory cytokines like IL-1β, IL-6, and TNF-α. The process of inflammation further induces oxidative stress, as immune cells, such as neutrophils, release large amounts of ROS through the NADPH oxidase pathway (Sun et al., 2023). In this study, Campesterol Derivatives and Piroxicam exhibited antioxidant properties by reducing MDA levels and increasing antioxidant molecules like thiol, SOD, and GPx, confirming their potential in combating oxidative stress.

Earlier research has shown that Campesterol Derivatives decreased collagen-based arthritis in mice and attenuated the inflammatory response in lipopolysaccharide-mediated fibroblast synoviocytes (Chaudhary et al., 2023). Additionally, sesquiterpene lactone Budlein A exhibited anti-inflammatory and analgesic effects in antigen-based arthritis in rats. In our study, Campesterol Derivatives significantly reduced paw thickness and arthritis scores, indicating their potential as an enduring antiarthritis agent for mitigating RA-associated stress.

To evaluate the antiarthritic and anti-inflammatory effects of Campesterol Derivatives in induced arthritic rats, specific conditions were established to ensure reliable results. The rats were divided into different groups based on their weight and received various treatments, including a normal control group, disease control group, piroxicam treatment group, high-dosage Campesterol Derivative group, and low-dosage Campesterol Derivative group (Ayati et al., 2019). The rats were observed over 28 days, and it was found that CFA-induced arthritis led to inflammation, synovial hyperplasia, bone deformation, cartilage degradation, edema, and impaired joint function in the control group (Arora et al., 2022). The use of campesterol derivatives and Piroxicam helped attenuate arthritic development, indicating their potential as therapeutic agents for RA (Nazir et al., 2023).

The campesterol derivatives demonstrated anti-inflammatory, analgesic, and antioxidant effects, making them a promising candidate for treating RA (Najm et al., 2020). The study revealed their potential in ameliorating arthritic symptoms and reduce oxidative stress. These findings highlight the therapeutic value of Campesterol Derivatives in managing the challenging manifestations of RA (Elshabrawy et al., 2015). The study’s limitations include the use of Sprague Dawley rats, which may not fully represent human rheumatoid arthritis, a small sample size, and a lack of detailed mechanistic insights. Additionally, the evaluation of oxidative stress markers was limited, and potential side effects of Campesterol Derivatives were not extensively investigated (Fraile et al., 2012). The short treatment duration and the absence of comparison with other treatments are also notable limitations. Despite these drawbacks, the study provides valuable insights into the drug’s anti-inflammatory and analgesic effects, warranting further research and clinical trials to validate its potential for rheumatoid arthritis treatment (Nirmal et al., 2012).

## 5 Conclusion

This research study focused on assessing the potential of Campesterol Ester Derivatives as a treatment for rheumatoid joint pain (RA). The study comprehensively explored the complexities of RA, its impact on patients, and the effectiveness of various treatments, including Campesterol Ester Derivatives and conventional therapies like DMARDs. The research meticulously examined the material and methods used, conducting detailed analyses of compound preparation, experimentation conditions, antiarthritic activity, and the assessment of various parameters. TNF-α & IL-6 mRNA expression levels were also studied, providing deeper insights into the therapeutic effects. Through a series of experiments on rats with CFA-induced arthritis, the study compared the effects of Campesterol Ester Derivatives and piroxicam. The results revealed that Campesterol Ester Derivatives exhibited significant anti-arthritis impacts, leading to paw oedema reduction and improved disease conditions. The findings of this research paper hold promise for future advancements in RA treatment and contribute valuable knowledge to the field. By validating the efficacy of Campesterol Ester Derivatives, this study opens up new possibilities for enhancing the quality of life for individuals suffering from RA. Further research and clinical trials are warranted to translate these discoveries into potential therapeutic options for RA patients.

## Data Availability

The raw data supporting the conclusion of this article will be made available by the authors, without undue reservation.
